# The Effects of Berberine and Palmatine on Efflux Pumps Inhibition with Different Gene Patterns in *Pseudomonas aeruginosa* Isolated from Burn Infections

**Published:** 2017

**Authors:** Seyed Sajjad Aghayan, Hamidreza Kalalian Mogadam, Mozhgan Fazli, Davood Darban-Sarokhalil, Seyed Sajjad Khoramrooz, Fereshteh Jabalameli, Somayeh Yaslianifard, Mehdi Mirzaii

**Affiliations:** 1. Department of Basic Sciences, Islamic Azad University, Damghan Branch, Damghan, Iran; 2. Faculty of Medicine, Shahroud University of Medical Sciences, Shahroud, Iran; 3. Department of Microbiology, Faculty of Medicine, Iran University of Medical Sciences, Tehran, Iran; 4. Medicinal Plants Research Center, Yasuj University of Medical Sciences, Yasuj, Iran; 5. Department of Microbiology Faculty of Medicine, Tehran University of Medical Sciences, Tehran, Iran; 6. Department of Microbiology, Faculty of Medicine, Alborz University of Medical Sciences, Karaj, Iran

**Keywords:** Berberine, Palmatine, *Pseudomonas aeruginosa*

## Abstract

**Background:**

Related Multidrug Resistance (MDR) to efflux pumps is a significant problem in treating infections caused by *Pseudomonas aeruginosa* (*P. aeruginosa*). Plant compounds have been identified as Pump Inhibitors (EPIs). In the current study, the potential effect of Berberine and Palmatine as EPIs were investigated on efflux pump inhibition through focusing on different gene patterns in *P. aeruginosa* isolated from burn infections.

**Methods:**

All isolates were collected and identified using the standard biochemical tests. Antimicrobial sensitivity was performed based on disk agar diffusion method for 12 antibiotics. MIC-MBC tests were also performed based on the broth microdilution method to detect synergistic relationship between ciprofloxacin, Berberine and Palmatine. Detection of *mexA, mexB, mexC, mexD, mexE, mexF and mexX* was conducted by PCR assay. Fisher’s Exact test and Logistic Regression were used as statistical tools.

**Results:**

A total of 60 *P. aeruginosa* isolates were collected. The highest and lowest levels of resistance were found to be respectively against clindamycin and tigecycline. Comparing the MIC with MBC distribution, it was found that Berberine and Palmatine lower the MIC-MBC level of ciprofloxacin. The PCR results indicated that the highest frequency is about MexAB-OprM operon. The statistical analysis among different gene patterns of efflux pumps showed that there were no significant relationships between the effectiveness of Berberine and Palmatine (p>0.05).

**Conclusion:**

It can be speculated that Berberine and Palmatine both act as EPIs and can be used as auxiliary treatments with the purpose of increasing the effect of available antibiotics as well as decreasing the emergence of MDR bacteria. The efficiency of these combinations should be studied further under *in vivo* conditions to have a more comprehensive conclusion regarding this issue.

## Introduction

Burns are of major public health concern; in fact, burns are considered to be among the most frequent and devastating forms of trauma. In addition, the burn patients have unique predisposition to different infections 1,2; considering this, *Pseudomonas aeruginosa (P. aeruginosa)* is known as the most common source of burn infections 3. In addition, infections caused by this bacteria resist against the most antimicrobial agents; this could be due to the expression of different mechanisms by this bacteria enabling it to overcome the effects of the agents 4. It should be noted that efflux pumps, which are attributed to multidrug resistance *via* extruding various classes of antimicrobial agents, are among the main mechanisms 5–7. It is of clinical significance that the efflux systems in *P. aeruginosa* are best characterized with RND family 8. This family is composed of three parts, including the transporter, the linker, and the outer membrane pore 9. *P. aeruginosa* contains 12 RND efflux systems among which only the functions of six systems (*i.e*. MexAB-OprM, MexCDOprJ, MexEF-OprN, MexGHI-OpmD, MexJK and Mex-XY) have been confirmed 10.

A novel and promising approach to overcome the multidrug resistant bacteria is to improve the clinical performance of various antibiotics through employing Efflux Pump Inhibitors (EPIs) 11. So far, plants have been comprehensively explored as potential sources for extracting efficient antimicrobials 12. Berberine and Palmatine are well-known natural alkaloids found mainly in roots and rhizomes of *Berberis vulgaris* 13. It should be mentioned that Berberine has been traditionally used among local people as an antimicrobial agent, mainly because of its effects on various microbes (*e.g*. virus, bacteria, fungi and protozoans) 14–16. Although few studies have shown that Berberine could be phenotypically considered as EPIs on standard strain of *Staphylococcus aureus*, there has not been any study, to the best of our knowledge, investigating the effects of Berberine on *P. aeruginosa*. In comparison with Berberine, Palmatine is a newer alkaloid compound and also, its pump inhibitory effects on bacteria have not been studied yet 17,18. It should be taken into account that in most of the conducted studies, the antibacterial and pump inhibitory effects of Barberine and Palmatine on standard samples of bacteria have been focused on.

The present study aimed to determine and compare the effects of Berberine and Palmatine on efflux pump inhibition in *P. aeruginosa* isolated from burn infections. The present study was conducted on clinical samples of *P. aeruginosa* isolated from burn infections in order to advance our knowledge in this field. Considering the high diversity of RND family in *P. aeruginosa,* the genetic distribution of this family was also determined from the samples. Furthermore, the relationship between the determined genetic patterns and phenotypic effects of Berberine and Palmatine on efflux pump in *P. aeruginosa* was investigated in this work.

## Material and Methods

### Sample collection and identification

Samples were collected between March 2013 and October 2014 from several hospitals in Tehran, Iran. Sterile swabs were applied to collect wound specimens from registered patients. These isolates were then subcultured on BHI agar and identified by using biochemical tests, including gram staining, catalase, oxidase, Oxidative-Fermentative (OF) test, pigment production, and growth at 42°*C*^19^. The bacterial strains were preserved in trypticase soy broth.

### Antibiotic susceptibility testing

Antimicrobial sensitivity tests were performed on Mueller-Hinton agar based on Kirby-Bauer disk diffusion method and interpreted according to CLSI (Clinical and Laboratory Standards Institute) standard tables^20^. In the tests, *P. aeruginosa* ATCC27853 was used as the control. In this study, the following antibiotic disks (MAST, UK) were applied: cefepime (30 *μg*), ceftriaxone (30 *μg*), ciprofloxacin (5 *μg*), clindamycin (2 *μg*), cotrimoxazole (25 *μg*), erythromycin (15 *μg*), gentamycin (120 *μg*), imipenem (10 *μg*), kanamycin (30 *μg*), rifampicin (5 *μg*), tetracycline (30 *μg*) and tigecycline (15 *μg*).

### Determination of MIC and MBC

The Minimum Inhibition concentration (MIC) and minimum bactericidal concentration (MBC) for the bacterial strains were determined using the broth microdilution method in 96-well microtiter plates which is based on the CLSI guidelines 20. After incubating the samples for 18–24 *hr* at 37°*C*, the bacterial cultures were diluted to a turbidity of 0.5 McFarland (1.5×10^8^ *CFU/ml*); and then, these cultures were further diluted in saline solution to obtain an inoculum of 5×10^5^ *CFU/well* in a final volume of 100 *ml*. The plates were incubated aerobically at 37°*C* for 18–24 *hr*. The MIC is defined as the lowest concentration from which there is no visible growth after incubation at 37°*C* for 18–24 *hr*. To determine the MBC, 5 *μl* of each well with no visible growth of bacterial was separately cultivated on Mueller-Hinton agar medium. After incubating these samples at 37°*C* for 18–24 *hr*, the extract with the lowest concentration and no growth of bacteria (with 99% precision) was reported as MBC concentration.

To verify the synergistic activity of ciprofloxacin with Berberine and Palmatine, the MIC and MBC of ciprofloxacin (512 *mg/ml*) alone was compared with ciprofloxacin along with Berberine or Palmatine (2000 *μg/ml*). Berberine and Palmatine were purchased from Sigma Aldrich Co, St. Louis, MO and used in the present study.

### DNA extraction and PCR assay

*P. aeruginosa* genomic DNA was extracted using the PrimePrep Genomic DNA isolation kit (GENETBIO). The efflux pump genes were determined by PCR using specific primers shown in table 1. The PCR reaction was conducted in a final volume of 25 *μl*; and also, the following parameters were taken into account in this reaction: PCR buffer (10×) 2.5 *μl*, MgCl2 (50 *mM*) 0.75 *μl*, dNTPs (10 *mM*) 1 *μl*, forward and reverse primers (10 *pmol/μl*) 1 *μl*+ 1 *μl*, Taq DNA polymerase (5 *U/μl*) 1 *μl*, distilled water 16.75 *μl* and template DNA 1 *μl*. The PCR products were analyzed using electrophoresis (100 *v*, 45 *min*) in gels composed of 1.5% agarose stained with DNA staining dye and visualized under specific UV light.

**Table 1.** Primers used for PCR

**Gene**	**Primer sequence (5′ to 3′)**	**Product size**	**Annealing temp**
***mexA***	F-CCTGCTGGTCGCGATTTCGG	312 *bp*	64.1
	R-CCAGCAGCTTGTAGCGCTGG		
***mexB***	F-GTGTTCGGCTCGCAGTACTC	224 *bp*	62.5
	R-AACCGTCGGGATTGACCTTG		
***mexC***	F-TTGGCTATGGCCATCGCGTT	371 *bp*	60.5
	R-ATCGAAGTCCTGCTGGCTGA		
***mexD***	F-CCCGAAATTCCTTTACGCGAT	146 *bp*	59.5
	R-CGCATTGGTGAAGTCGTTGA		
***mexE***	F-ATCCCACTTCTCCTGGCGCT	240 *bp*	62.5
	R-GGTCGCCTTTCTTCACCAGT		
***mexF***	F-AAGTACGCCGACATTCAGGA	131 *bp*	58.4
	R-TGATGATGTTCTGGGTCTGCT		
***mexX***	F-GCGATGCGGATTGCGGAACA	792 *bp*	62.5
	R-TGGTCGCCCTATTCCTGCTG		

### Statistical analysis

Statistical analysis was performed using Statistical Package for the Social Sciences software (SPSS Software V. 19) with Fisher’s Exact test as well as Logistic Regression; and also, p<0.05 was considered to show the statistically significant results.

## Results

In this study, a total of 60 *P. aeruginosa* isolates were collected from the burn infections. Bacterial identification was performed using the standard biochemical tests. Table 2 shows the results of the antimicrobial susceptibility patterns. As can be seen from the table, *pseudomonas aeruginosa* isolates showed the highest rate of resistance to clindamycin and kanamycin by 93.33 and 91.67%, respectively; whereas, the resistance rate for tigecycline was found to be 6.67%. In addition, an intermediate sensitivity to imipenem was found to be 81.67%.

**Table 2.** Antimicrobial susceptibility pattern

**Antibiotic**	**Susceptible (%)**	**Intermediate (%)**	**Resistant (%)**
**Cefepime**	13.33	-	86.67
**Ceftriaxone**	11.67	-	88.33
**Ciprofloxacin**	10	-	90
**Clindamycin**	6.67	-	93.33
**Cotrimoxazole**	10	-	90
**Erythromycin**	6.67	3.33	90
**Gentamycin**	66.67	-	33.33
**Imipenem**	13.33	81.67	5
**Kanamycin**	8.33	-	91.67
**Rifampicin**	11.67	-	88.33
**Tetracycline**	53.33	8.33	38.34
**Tigecycline**	93.33	-	6.67

The MIC and MBC distribution of ciprofloxacin, Berberine, Palmatine, ciprofloxacin+Berberine and ciprofloxacin+Palmatine on the ciprofloxacin resistance isolates are indicated in table 3. The MIC of ciprofloxacin in combination with 1/2 MIC of Berberine and Palmatine were separately determined by broth microdilution. As shown in the table, the highest ranges of MIC for ciprofloxacin were 128 *mg/ml* and 64 *mg/ml*. In addition, the highest ranges of MIC for Barberine in the samples were 250 *μg/ml* (54.17%) and 125 *μg/ml* (33.33%). Regarding the MIC for Palmatine, the highest ranges were found to be 500 *μg/ml* (62.5%) and 250 *μg/ml* (37.5%). By analyzing the mean±SD of MIC for ciprofloxacin and Barberine, and Palmatine, there was 1.1±0.2 log growth reduction in 91.67% and 83.34% of the isolates. Furthermore, the results of the mean±SD MBC analyses for ciprofloxacin and Barberine and Palmatine indicated 1±0.2 log growth reduction in 91.66 and 87.75% of the isolates.

**Table 3.** Distribution of MIC and MBC (%)

	**1**	**1/2**	**1/4**	**1/8**	**1/16**	**1/32**	**1/64**	**1/128**	**1/256**	**1/512**	**1/1024**
**Cip_MIC_**			4.16	41.67	41.67	12.5					
**Cip_MBC_**		16.66	41.67	41.67							
**Ber_MIC_**		12.5	54.17	33.33							
**Ber_MBC_**	41.67	50	8.33								
**Pal_MIC_**		62.5	37.5								
**Pal_MBC_**	87.5	12.5									
**Cip+Ber_MIC_**				8.33	58.34	33.33					
**Cip+Ber_MBC_**		8.34	45.83	45.83							
**Cip+Pal_MIC_**			4.16	12.5	66.67	16.67					
**Cip+Pal_MBC_**		12.5	41.67	45.83							

* Ciprofloxacin (512 *mg/ml*)

* Berberine and palmatine (2000 *μg/ml*)

**Figure 1.**
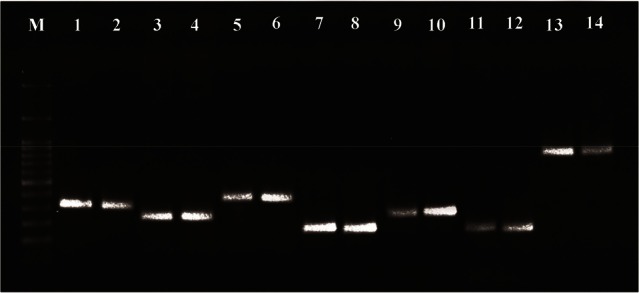
PCR products of Pseudomonas RND family efflux pump genes. Line M: DNA size marker (SMOBIO, DM2301100 *bp*-3000 *bp*), line 1–2: *mexA* (312 *bp*), line 3–4: *mexB* (224 *bp*), line 5–6: *mexC* (371 *bp*), line 7–8: *mexD* (146 *bp*), line 9–10: *mexE* (240 *bp*), line 11–12: *mexF* (131 *bp*) and line 13–14: *mexX* (792 *bp*).

Based on the results of PCR efflux pump genes, the highest frequency belonged to *mexB* with 100%. The frequencies of other genes were 59 strains of *mexA* (98.3%), 59 strains of *mexD* (98.3%), 59 strains of *mexF* (98.3%), 58 strains of *mexX* (96.6%), 54 strains of *mexC* (90%) and 54 strains of *mexE* (90%) (Figure 1). The highest frequency was due to MexAB-OprM operon. The result of Fisher’s Exact test showed that there were no significant relationships between each gene profile of efflux pumps per se in terms of the effectiveness of Berberine and Palmatine. The comparison between the MIC mean±SD of Berberine and Palmatine with respect to the ciprofloxacin efficiency was not significant. Moreover, the Logistic Regression revealed that there were not any significant relationships between the cumulative frequency of gene templates of efflux pumps in terms of the effectiveness of Berberine and Palmatine’s impact.

## Discussion

Despite medical achievements, infection is still the main cause of death among the burn patients. *P. aeruginosa* is one of the commonest causes of infection in burn wounds. It is important to identify multidrug resistance pattern in isolates. In the current study, the highest level of resistance was found to be against clindamycin, kanamycin, ciprofloxacin, cotrimoxazole and erythromycin; whereas, the lowest level of resistance was against tigecycline, gentamicin and tetracycline. These results were not consistent with the reports of previously conducted studies 21,22. It could possibly be related to different sources of infection, bacterial strains and treatment regimens being applied in various regions.

One of the important problems of treating the infections caused by *P. aeruginosa* is that the bacteria are multidrug resistant; a main resistance factor is the existence of efflux pumps, especially the RND family. In the present study, the highest frequency was found for MexAB-OprM operon, which is in line with the reports of previous studies 23,24. The prior reports indicated that MexAB-Oprm operon is important in the natural resistance against fluoroquinolones and pathogenicity in terms of the structure of gene expression in *P. aeruginosa* 25,26.

Comparing the results of antimicrobial susceptibility patterns and MIC ciprofloxacin in the present work with those of previous studies, it was found that the strains are more resistant against ciprofloxacin than the other agents 27,28; it should be noted that this might be caused by the indiscriminate use of antibiotics in burn injury centers and also the diversity in the source separating bacterium from patient. One of the most important strategies in developing resistance against ciprofloxacin is to use efflux pumps 29; it can be implied that the frequency of genes related to efflux pumps in the studied samples was high.

The EPIs can be used as auxiliary treatments with the purpose of increasing the effect of available antibiotics and decreasing the emergence of multidrug resistance bacteria. Several EPIs with the capability to affect the pumps of RND family have been found and reported in previous studies 30. It should be noted that none of the EPIs has been clinically developed. The hybrid plants could be produced to inhibit the efflux-dependent resistance. Therefore, identifying and using bio-active phytochemicals existing in the extract of plants along with the activity of EPIs can result in a better inhibition of efflux pumps 31.

Berberine and Palmatine are among the bio-active phytochemicals existing in the extract of Berberis Vulgaris with the activity of EPIs 18. The MIC results of Berberine for *P. aeruginosa* (250–1000 *μg/ml*) was consistent with the results of a study conducted by Tegos *et al*, (500 *μg/ml*) and Jian-ling *et al*, (2000 *μg/ ml*) 32,33. A slight difference in the effect of Berberine on *P. aeruginosa*, compared with the corresponding results of other studies, could be due to the large number of clinical samples taken and analyzed in the current study. The MIC range of Palmatine (500–1000 *μg/ml*) was found to be lower than that of Berberine, which could be due to their difference in terms of chemical structures (p≤0.05). To the best of our knowledge, there is no similar study in which the effect of Palmatine on *P. aeruginosa* is focused on. Separately, comparing the effect range of ciprofloxacin alone and with the effect range of ciprofloxacin+Berberine and ciprofloxacin+ Palmatine, it was found that Berberine and Palmatine decreased up to 1±0.2 logarithm the MIC level in 91.67 and 83.34% of strains, respectively. Berberine and Palmatine also decreased up to one logarithm the MBC level in 91.66 and 87.5% of strains, respectively. 1±0.2 logarithm decrease in the growth of *P. aeruginosa* is an indication of the bacteriostatic effect of Berberine and Palmatine on the clinical samples. Therefore, it can be implied that they cannot be used as the only antibiotics for treating purposes; in other words, they should be used as a supplement to increase the synergy, contributing to an increased effect of antibiotics.

Based on the results of Fisher’s Exact test and Logistic Regression model among different gene patterns of efflux pumps, no significant relationship was observed regarding the effectiveness of Berberine and Palmatine. In other words, Berberine and Palmatine probably have inhibiting impacts on different gene patterns of efflux pumps influenced by the expression of several genes; hence, there is a need for more studies relating to this issue. Considering the diverse effects of Berberine and Palmatine on different genes of efflux pumps, it can be implied that there were not any significant relationships between the subjects of different gene patterns of efflux pumps in terms of the effectiveness of such medicines on the inhibition of efflux pumps.

## Conclusion

In this work, the effects of Berberine and Palmatine on efflux pump inhibition in *P. aeruginosa* isolated from burn infections were determined and compared with each other. Different studies have been conducted on the ability of herbal extracts to decrease antibiotic resistance. Nevertheless, studies conducted on bio-active phytochemicals (*e.g*. Berberine and Palmatine) as EPIs are at the initial *in vitro* stage. In addition, it was found that Berberine and Palmatine decreased up to 1±0.2 log of the MIC level in the majority of clinical strains, which can be considered as an indication of the bacteriostatic effects of these compounds. Therefore, Berberine and Palmatine probably can be used as supplements to enhance the synergistic effects of antibiotics. However, the conditions of clinical applications of EPIs have not been well clarified yet. It is essential that the efficiency of these combinations be studied under *in vivo* conditions. Comparing RND family gene patterns with Berberine and Palmatine pump inhibitory effect, it is necessary to investigate the effect of Berberine and Palmatine in the expression of RND family genes.
